# E-cadherin expression in macrophages dampens their inflammatory responsiveness *in vitro,* but does not modulate M2-regulated pathologies *in vivo*

**DOI:** 10.1038/srep12599

**Published:** 2015-07-31

**Authors:** Jan Van den Bossche, Damya Laoui, Thomas Naessens, Hermelijn H. Smits, Cornelis H. Hokke, Benoît Stijlemans, Johan Grooten, Patrick De Baetselier, Jo A. Van Ginderachter

**Affiliations:** 1 Laboratory of Myeloid Cell Immunology, VIB, Brussels, Belgium; 2 Laboratory of Cellular and Molecular Immunology, Vrije Universiteit Brussel, Brussels, Belgium; 3 Department of Biomedical Molecular Biology, Ghent University, Ghent, Belgium; 4 Department of Parasitology, Leiden University Medical Centre, Leiden, The Netherlands

## Abstract

IL-4/IL-13-induced alternatively activated macrophages (M_(IL-4/IL-13)_, AAMs or M2) are known to express E-cadherin, enabling them to engage in heterotypic cellular interactions and IL-4-driven macrophage fusion *in vitro*. Here we show that E-cadherin overexpression in Raw 264.7 macrophages inhibits their inflammatory response to LPS stimulation, as demonstrated by a reduced secretion of inflammatory mediators like interleukin (IL)-6, *tumor necrosis factor* (TNF) and nitric oxide (NO). To study the function of E-cadherin in M_(IL-4/IL-13)_ macrophages *in vivo*, we generated macrophage-specific E-cadherin-deficient C57BL/6 mice. Using this new tool, we analyzed immunological parameters during two typical AAM-associated Th2-driven diseases and assessed Th2-associated granuloma formation. Although E-cadherin is strongly induced in AAMs during *Taenia crassiceps* helminth infections and allergic airway inflammation, its deletion in macrophages does not affect the course of both Th2 cytokine-driven diseases. Moreover, macrophage E-cadherin expression is largely redundant for granuloma formation around *Schistosoma mansoni* ova. Overall, we conclude that E-cadherin is a valuable AAM marker which suppresses the inflammatory response when overexpressed. Yet E-cadherin deletion in macrophages does not affect M_(LPS+IFNγ)_ and M_(IL-4)_ polarization *in vitro*, nor *in vivo* macrophage function, at least in the conditions tested.

Depending on the microenvironment, macrophages are polarized to different subsets which have been broadly classified as M1 and M2. Classically activated macrophages (CAMs or M1) are induced by Th1 inflammatory cytokines, such as IFN-γ, and by microbial or endogenous danger-associated molecules. According to the latest nomenclature guidelines, CAMs or M1 are now classified as M_(LPS)_, M_(IFNγ)_ or M_(LPS+IFNγ)_, depending on the activators used to generate them^1^. These inflammatory macrophages produce cytokines like IL-1β, IL-6, IL-12 and TNF and express high levels of inducible nitric oxide synthase (iNOS)^2^, which makes them potent effector cells to combat microorganisms and potentially also tumor cells. Macrophages are also activated by anti-inflammatory mediators, including the Th2 cytokines interleukin-4 (IL-4) and IL-13, IL-10, transforming growth factor-β (TGF-β), glucocorticoids, and immune complexes. While all these types of ‘non-M1’ macrophages are often grouped under the generic term M2^3^, this nomenclature is often indistinct and confusing^1^,^4^. Therefore, the formerly so-called IL-4 (and/or IL-13)-induced alternatively activated macrophages (AAMs) are now termed M_(IL-4)_ or M_(IL-4/IL-13)_^1^. M_(IL-4/IL-13)_ inhibit Th1/M1-driven inflammatory responses, promote Th2 responses, induce angiogenesis and wound repair, and can be immunosuppressive^5^. However, it is not fully-understood how these diverse functions are regulated at the molecular level.

We previously identified E-cadherin as a novel IL-4/IL-13-induced, STAT6/polyamine-dependent marker for M_(IL-4/IL-13)_^6^,^7^. E-cadherin co-localizes with β- and p120-catenin at the cell surface, enabling M_(IL-4/IL-13)_ macrophages to undergo homotypic adhesive interactions, leading to cell fusion upon IL-4 treatment *in vitro.* Macrophages still fuse in the absence of E-cadherin, but the number of nuclei in each giant cell and their size is reduced^6^. In fact, different IL-4-induced molecules, including E-cadherin, DC-STAMP and TREM-2, need to cooperate to induce fusion-competent macrophages^8^,^9^. Furthermore, E-cadherin^+^ macrophages engage in heterotypic interactions with KLRG1^+^ and CD103^+^ cells *in vitro*. Upon ligation, KLRG1 inhibits TCR signaling and NK cytotoxicity which could be a way for E-cadherin^+^ cells to impair inflammatory immune responses^10^. Interestingly, CD103 is found on major mediators of the immune response, such as DC and T cell subsets^11^,^12^. Hence, E-cadherin might serve to bring these cells in closer contact with macrophages, thereby potentially influencing their retention and phenotype during polarized Th2 responses.

Besides their function in cell adhesion, E-cadherin and its associated catenins may modulate intracellular signaling molecules, including β-catenin/Wnt^13^, phosphatidylinositol 3-kinase (PI3K)^14^, Rho-family GTPases^15^ and NFκB^16^,^17^,^18^. As such, E-cadherin reduces the inflammatory response in keratinocytes and epithelial cells. Furthermore, KLRG1 engagement of E-cadherin on DCs lowers their secretion of inflammatory cytokines, thereby exerting immunosuppressive effects^19^. Hence, it is conceivable that the E-cadherin/catenin complex might exert similar activities in macrophages and could contribute to the anti-inflammatory character and immunoregulatory capacity of alternatively activated M_(IL-4/IL-13)_ during Th2 responses.

We demonstrate here that E-cadherin overexpression indeed suppresses the secretion of inflammatory mediators. Yet, while E-cadherin is a valuable marker for polarized Th2 responses and M_(IL-4/IL-13)_, its macrophage-specific deletion has no major *in vivo* effects on macrophage activity during Th2 responses, nor on cell fusion during *Schistosoma mansoni* granuloma formation.

## Results

### E-cadherin expression in Raw264.7 macrophages reduces their inflammatory phenotype upon stimulation

**Figure 1:**
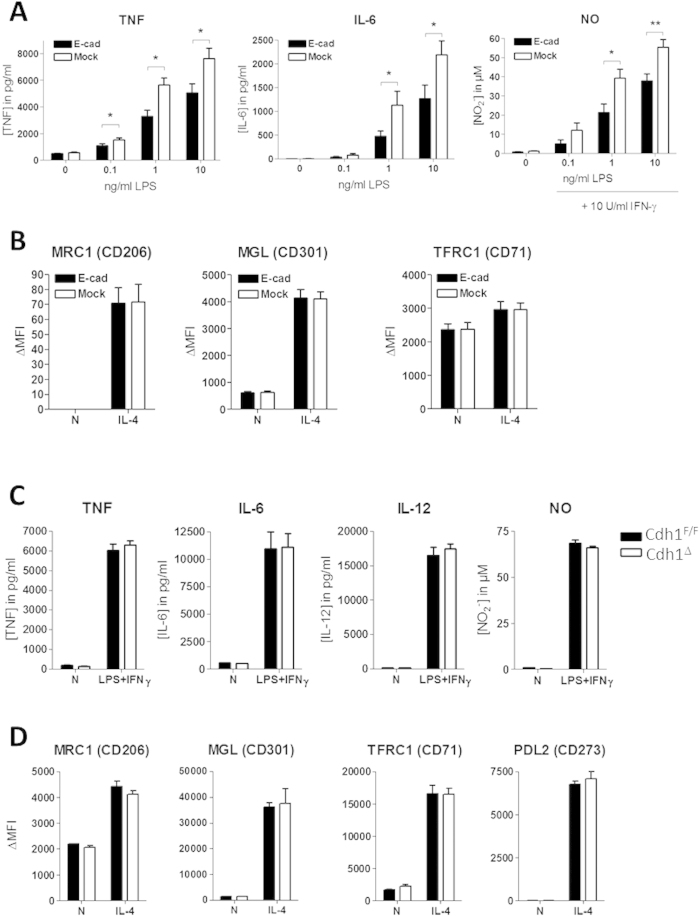
E-cadherin-overexpressing Raw264.7 macrophages display reduced inflammatory responses. Raw-E-cadherin and Raw-Mock transfectants (n = 4) were left untreated or were stimulated for 24 h with (**A**) indicated concentrations LPS (+ IFNγ) or (**B**) IL-4. Similarly, BMDM from naive Cdh1^Δ^ and Cdh1^F/F^ mice (n = 3) were treated with (**C**) 10 ng/ml LPS + 10 U/ml IFNγ or (**D**) with IL-4. M_LPS(+IFNγ)_ and M_IL-4_ polarization was assessed by measuring the secretion of IL-6, IL-12, TNF and NO or by determining the IL-4-induced surface expression MRC1, MGL, TFRC1 and PDL2, respectively. IL-12 secretion was not detected in Raw264.7 cells and these cells did not show IL-4-induced PDL2 upregulation. Data represent the mean ± SEM of 4 individual Raw264.7 clones (**A**,**B**) or BMDM from 3 individual mice (**C**,**D**). ΔMFI = [median fluorescence intensity]_positive staining_ − [median fluorescence intensity]_isotype control_. *P < .05; **P < .01; ***P < .001

Besides its well-described role as an adhesive molecule, E-cadherin influences inflammatory signaling pathways such as NF-κB and thereby inhibits the inflammatory activation of various cell types^16^,^17^,^18^. As such, E-cadherin is now emerging as a potentially important immunological regulator^20^,^21^. To test the effect of E-cadherin expression on inflammatory cues in macrophages, we generated four independent E-cadherin over-expressing Raw264.7 transfectants and four E-cadherin-negative mock transfectants (Figure S1A). LPS dose-dependently induced TNF and IL-6 secretion by Raw264.7 macrophages after 24 h stimulation and this response was significantly lowered in E-cadherin-overexpressing transfectants (Fig. 1A). Even at the highest LPS concentration, E-cadherin was still able to inhibit LPS-induced production of inflammatory cytokines. Moreover, NO induction by IFNγ and increasing concentrations of LPS was reduced in macrophages that express E-cadherin. Conversely, E-cadherin overexpression did not affect M_(IL-4)_ polarization as illustrated by the unaltered IL-4-induced surface expression of Macrophage Mannose Receptor (MMR, CD206), Macrophage Galactose-type C-type Lectin (MGL, CD301), and Transferrin Receptor 1 (CD71) (Fig. 1B). Together, these data show that E-cadherin actively participates in down-tuning the macrophage inflammatory responsiveness when overexpressed, a feature that would be consistent with its predominant expression in anti-inflammatory M_(IL-4)_ macrophages.

In naive conditions, primary macrophages express very low levels of E-cadherin^6^. To assess whether E-cadherin deletion at baseline would affect subsequent M_(LPS+IFNγ)_ polarization in primary macrophages, E-cadherin-deficient bone marrow-derived macrophages (LysM-cre x Cdh1^F/F^, termed Cdh1^Δ^) were generated and treated with LPS+IFNγ. These cells displayed a similar M_(LPS+IFNγ)_ polarization as their WT (Cdh1^F/F^) counterparts (Fig. 1C) *in vitro*. Accordingly, we did not observe differences in survival between Cdh1^Δ^ and Cdh1^F/F^ mice upon LPS-induced sepsis as typical M1 response *in vivo* (Figure S1B). Moreover, bone marrow-derived macrophages from Cdh1^Δ^ and Cdh1^F/F^ mice displayed similar M_(IL-4)_ polarization (Fig. 1D), phagocytosis and autophagy (Figure S1C,D). Hence, only high levels of E-cadherin at baseline alter the responsiveness to a subsequent inflammatory insult.

### Cdh1^Δ^ and control mice are equally susceptible to Taenia crassiceps infection

**Figure 2:**
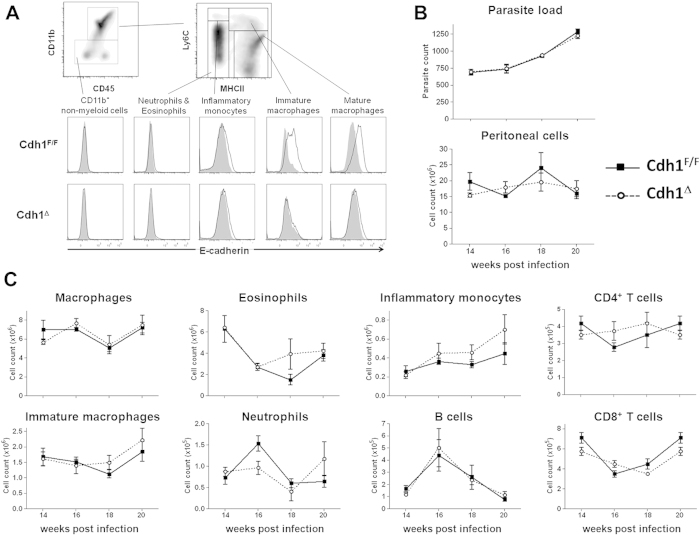
Cdh1^Δ^ mice are equally susceptible to *Taenia crassiceps* infection. (**A**) Freshly isolated peritoneal cells from *T. crassiceps* infected Cdh1^F/F^ and Cdh1^Δ^ mice (14 weeks p.i.) were subjected to multicolour FACS analysis. Myeloid and non-myeloid (G1) cells were first gated based on CD11b expression. Within the CD11b^+^ myeloid cell gate, Ly6C-MHC II staining discriminates between Ly6C^int^/MHC II^neg^ eosinophils and neutrophils, Ly6C^high^/MHC II^neg^ inflammatory monocytes, Ly6C^hi^/MHC II^pos^ immature macrophages and Ly6C^low^/MHC II^high^ mature macrophages. The histograms show an overlay of isotype staining (grey, filled) and anti-E-cadherin staining (bold line) on these gated peritoneal cell types from *T. crassiceps* infected Cdh1^F/F^ (top) and Cdh1^Δ^ (bottom) mice. (**B**) Parasite load (top) and total leukocyte cell count (bottom) is similar in the peritoneal cavities of Cdh1^Δ^ and Cdh1^F/F^ infected mice at all indicated time points. (**C**) Peritoneal cells from *T. crassiceps* infected Cdh1^F/F^ and Cdh1^Δ^ mice were isolated at indicated time point and subjected to multicolour FACS analysis and all leukocyte populations were gated as shown in Figure S2. The absolute number of macrophages, eosinophils, inflammatory monocytes, immature macrophages, neutrophils, B cells and CD4+ and CD8+ T cells in the peritoneal cavities of *T. crassiceps* infected Cdh1^F/F^ and Cdh1^Δ^ mice (average ± SEM of 5 mice per group per time point) is shown.

**Figure 3:**
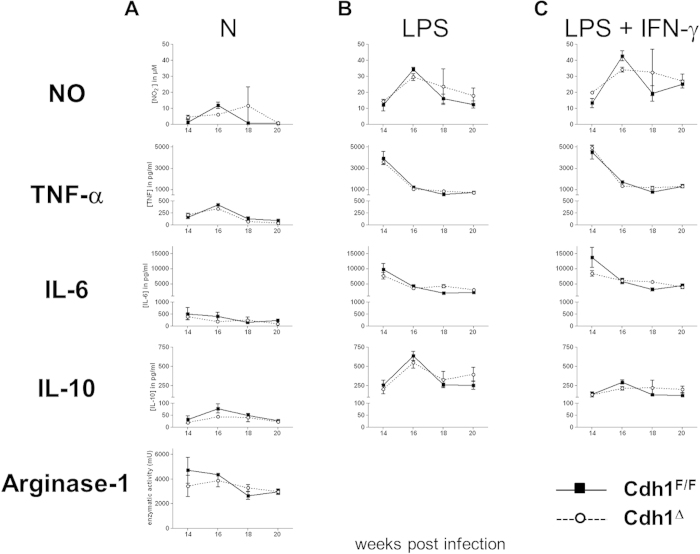
Peritoneal macrophages from Cdh1^F/F^ and Cdh1^Δ^ *T. crassiceps*-infected mice display a similar activation status. (**A**) Peritoneal macrophages obtained from Cdh1^F/F^ and Cdh1^Δ^ mice at different time points after infection display similar arginase activity and spontaneously secrete equal levels of NO, TNF, IL-6 and IL-10 upon 24 h *in vitro* culture. Additionally, these *in vivo*-elicited cells secrete similar amounts of NO, TNF, IL-6 and IL-10 upon (**B**) LPS and (**C**) LPS+IFN-γ stimulation. Values are the mean ± SEM of 5 mice per group per time point.

Next, we set out to investigate the immunoregulatory role of E-cadherin in M_(IL-4/IL-13)_ macrophages *in vivo* by assessing the course of Th2- and M_(IL-4/IL-13)_-associated diseases in Cdh1^Δ^ versus Cdh1^F/F^ mice. *Taenia crassiceps* helminths initially induce a Th1 response which becomes highly Th2 polarized after 4 weeks of infection, resulting in the accumulation of M_(IL-4/IL-13)_ macrophages that are essential for the *in vivo* maintenance of the parasite^22^. Hence, an alteration of the macrophage activation state, possibly triggered by the absence of E-cadherin, might impact the course of this infectious disease. In the peritoneal exudate cells from 14 weeks infected WT mice, surface expression of E-cadherin was confined to CD11b^hi^Ly6C^hi^MHC-II^hi^ immature macrophages and CD11b^hi^Ly6C^lo^MHC-II^hi^ mature macrophages. This expression is blunted in Cdh1^Δ^ mice, illustrating the efficiency of the gene deletion in macrophages (Fig. 2A). However, parasite load as well as the total cell counts were similar in the peritoneal cavities of Cdh1^Δ^ and Cdh1^F/F^ infected mice at all time points measured (Fig. 2B). In addition, the peritoneal cellular composition, gated as shown in Figure S2, is largely comparable between both mouse strains (Fig. 2C), excluding a major impact of E-cadherin expression in macrophages on the course of this disease. We next focussed specifically on the activation state of peritoneal macrophages from late-stage infected Cdh1^F/F^ and Cdh1^Δ^ mice. Macrophages from both strains had a similar arginase activity and spontaneously secreted comparable levels of NO, TNF, IL-6 and IL-10 upon *in vitro* culture (Fig. 3A), suggesting no major differences in the inflammatory status of these *in vivo*-elicited populations. Finally, we assessed the LPS responsiveness of the E-cadherin^+^ Cdh1^F/F^ macrophages versus the E-cadherin^−^ Cdh1^Δ^ macrophages from infected mice. As illustrated in Fig. 3B,C, these *in vivo*-elicited cells produced similar amounts of NO, TNF, IL-6 and IL-10, both spontaneously and upon LPS or LPS+IFN-γ stimulation *in vitro*. In conclusion, during a helminth infection that strongly polarizes the macrophages towards M_(IL-4/IL-13)_, the lack of E-cadherin in these cells is not sufficient to alter their activation state and to have an impact on the outcome of the disease.

### Cdh1^Δ^ and control mice are equally susceptible to allergic airway inflammation, but Cdh1^Δ^ mice display lower B cell counts in their BAL

**Figure 4:**
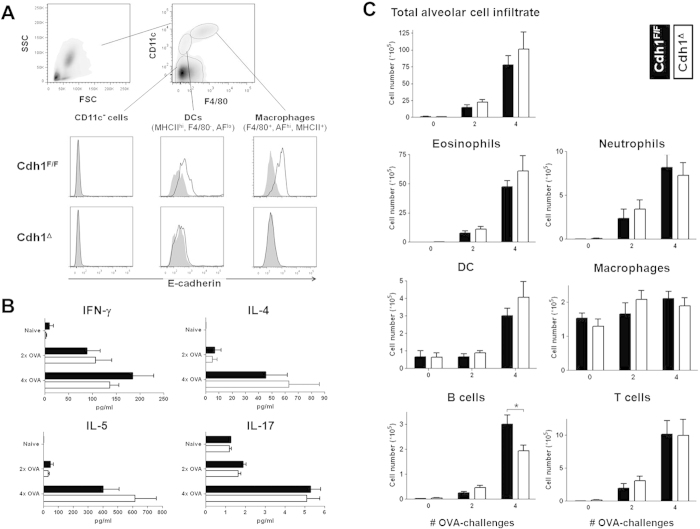
Cdh1^Δ^ mice are equally susceptible to ovalbumine-induced allergic airway inflammation. Allergic asthma was induced in Cdh1^F/F^ and Cdh1^Δ^ animals by ovalbumin sensitization with Alum adjuvant, followed by 2 (2× OVA, short term protocol) or 4 (4× OVA, long term protocol) ovalbumin aerosols. (**A**) Cells in the bronchoalveolar lavage (BAL) were stained with anti-CD11c, anti-F4/80, anti-MHC II and anti-E-cadherin or isotype control and analysed via FACS. Within gated CD11c^high^ BAL, a distinction was made between autofluorescence (AF)^low^ MHC II^high^ DC and AF^high^ F4/80^+^ alveolar macrophages and histogram overlays of isotype staining (grey, filled) and anti-E-cadherin staining (bold line) are shown for 4× OVA-challenged Cdh1^F/F^ (top) and Cdh1^Δ^ (bottom) mice. (**B**) IFN-γ , IL-4, IL-5 and IL-17 concentrations were measured in the BAL fluid of Cdh1^F/F^ (black bars) and Cdh1^Δ^ (white bars) mice. (**C**) Different leukocyte populations in the BAL of 2× and 4× ovalbumin-challenged Cdh1^F/F^ and Cdh1^Δ^ mice were gated as in Figure S3 and absolute cell counts are shown (average ± SEM of 5 mice per group per time point). *P < .05.

To further assess a potential role for macrophage E-cadherin during strongly polarized Th2-associated pathologies, we turned to a model of ovalbumine (OVA)-induced allergic airway inflammation. Sensitized mice were either challenged twice (short term protocol 2× OVA) or four times (long term protocol 4× OVA) with OVA allergen, during which E-cadherin-expressing alveolar macrophages (CD11c^high^ F4/80^+^ MHC-II^+^ autofluorescence (AF)^high^ cells) and DCs (CD11c^high^ F4/80^−^ MHC-II^hi^ AF^low^ cells) are induced (Fig. 4A for 4× OVA, data not shown for 2× OVA). Again, the E-cadherin expression was absent in Cdh1^Δ^ mice. While two ovalbumin challenges (2× OVA) were sufficient to induce an initial modest inflammatory response with faintly increased IL-4 and IL-5 levels in the bronchoalveolar lavage (BAL), the inflammation and associated secretion of IL-4, IL-5 and IL-17 were further increased by two additional ovalbumin challenges (4× OVA). However, no significant differences in BAL cytokine concentrations were observed between Cdh1^Δ^ and Cdh1^F/F^ control mice (Fig. 4B). In agreement with the similar cytokine profiles, Cdh1^Δ^ and Cdh1^F/F^ mice had comparable total BAL cell numbers (gated as shown in Figure S3) and a comparable recruitment of eosinophils, neutrophils and T cells to the alveolar space. Surprisingly, B cell counts were consistently lower in the BAL of Cdh1^Δ^ mice (Fig. 4C). Overall, deletion of macrophage (and DC) E-cadherin expression does not seem to affect the severity of ovalbumine-induced allergic airway responses.

### Cdh1^Δ^ mice display normal granuloma formation during schistosomiasis

**Figure 5:**
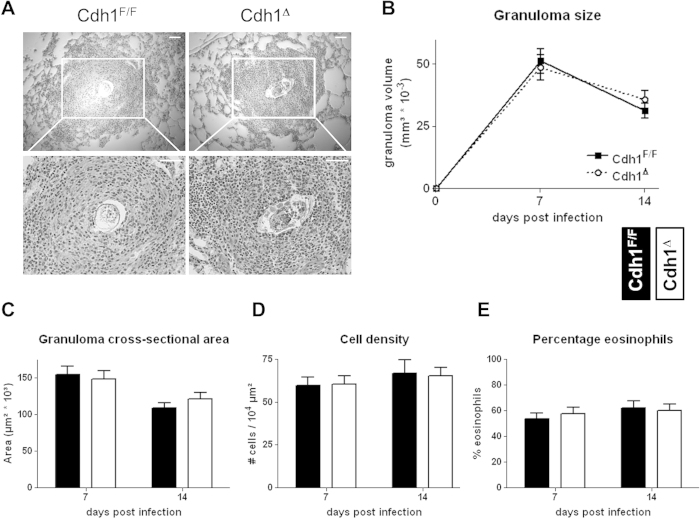
Cdh1^Δ^ mice display normal granuloma formation during schistosomiasis. (**A**) H&E stained lung sections from *Schistosoma mansoni* egg-challenged Cdh1^F/F^ and Cdh1^Δ^ mice containing a representative granuloma are shown at 10× (top) and 20× (bottom) magnification. (**B**) The granuloma size (calculated as V = 4/3*Π*r^3^), (**C**) the granuloma cross-sectional area, (**D**) the granuloma density (calculated as the amount of cells per 10^4^ μm^2^) and (**E**) the percentage eosinophils within each granuloma is shown after 7 and 14 days post egg challenge (average ± SEM, n = 5 mice per group per time point).

Earlier findings from our lab and others showed an involvement of E-cadherin in IL-4-mediated macrophage fusion leading to multinucleated giant cell formation *in vitro*^6^,^23^, but the *in vivo* relevance of these findings remained unaddressed. As a model system, we intravenously challenged Cdh1^Δ^ and Cdh1^F/F^ mice with *Schistosoma mansoni* eggs^24^, resulting in the induction of a polarized Th2 immune response and the formation of large pulmonary granulomas consisting mainly of eosinophils and macrophages (Fig. 5A). IL-4/IL-13-dependent formation of multinucleated giant cells is a hallmark of such granulomatous responses, suggesting a potential role for macrophage E-cadherin in this process^25^. However, no significant differences were found in granuloma size (Fig. 5B) nor cross-sectional area (Fig. 5C) between Cdh1^Δ^ and Cdh1^F/F^ mice at 7 and 14 days post challenge. In both mouse strains the granuloma size and area equally decreased from day 7 to day 14 post challenge. Moreover, the cell density within the granuloma (Fig. 5D) and its composition (as measured by the percentage eosinophils, Fig. 5E) was the same in both mouse strains. Overall, we have no evidence to conclude that macrophage E-cadherin expression contributes to granuloma formation in the lungs after intravenous *S. mansoni* egg challenge.

## Discussion

Parasitic helminths and allergens induce polarized Th2 responses, supporting alternative macrophage activation. While M_(IL-4/IL-13)_ macrophages are key regulators of these diseases^26^,^27^, discriminative surface markers allowing their identification and contributing to their function remained limited. Previously, we identified E-cadherin as a protein that associates with IL-4/IL-13-exposed mouse and human macrophages that can be employed as useful reporter of polarized Th2 responses and M_(IL-4/IL-13)_ macrophages^6^.

While E-cadherin clearly contributes to IL-4-driven macrophage fusion and the formation of multinucleated giant cells *in vitro*^6^,^23^, the *in vivo* significance of this observation remained unstudied so far. In this context, we here assessed E-cadherin’s contribution to granuloma formation during schistosomiasis *in vivo. Schistosoma mansoni* eggs are potent inducers of M_(IL-4/IL-13)_ macrophages, and granuloma formation around these ova is an important characteristic of schistosomiasis^28^,^29^. ‘Granuloma closure’, preventing the diffusion of parasitic substances to the surroundings, is an important event and cadherins are documented to be expressed during this process^30^. As such, macrophage E-cadherin could serve to bring granulomatous cells into close contact, allowing the formation a compactly packed cell mass around the schistosome egg. Nevertheless, an experimental intravenous challenge with *Schistosoma mansoni* eggs equally induced granuloma formation in the lungs of Cdh1^Δ^ and Cdh1^F/F^ mice. In addition, no differences in granuloma parameters could be observed between these mouse strains. Probably a wide range of fusiogenic molecules is involved during the compaction of granulomas, and deleting one (in this case E-cadherin) in one of the participating granulomatous cell types (here macrophages) is not enough to impair this process. Overall, we have no data to conclude that macrophage E-cadherin expression is fundamental for granuloma formation. Together our findings support a model whereby a complex collection of molecules interact to form multinucleated giant cells and granulomas, as suggested earlier^31^,^32^. Within this collection of participating molecules, redundancy might exist, as is demonstrated here for E-cadherin.

Besides its potential contribution to macrophage fusion, E-cadherin enables M_(IL-4/IL-13)_ macrophages to interact with KLRG1^+^ cells and to trap α_E_β_7_ integrin (CD103)-expressing cells *in vitro*^6^. Both KLRG1 and CD103 are E-cadherin ligands and are detected on major mediators of the immune system, including DC, NK and T cell subpopulations^11^,^12^,^21^,^33^,^34^. As such, E-cadherin on M_(IL-4/IL-13)_ macrophages might influence the retention of these cells in order to instruct their phenotype during Th2 immune responses. In parallel to its role during cell/cell interactions, the E-cadherin/catenin complex regulates inflammatory cascades in epithelial cells^16^,^18^ and thus might also modulate the phenotype of macrophages. In this context, possible effects of enhanced macrophage E-cadherin expression on NFκB, PI3K and β-catenin/Wnt signaling might modulate the macrophage activation status in a subtle manner. Indeed, these pathways are crucial to instruct the inflammatory phenotype of macrophages and are known to be modulated by E-cadherin and its different catenins in epithelial cells, keratinocytes, but also in tolerogenic DC^13^,^14^,^15^,^16^,^17^,^21^. Supporting this hypothesis, we showed here that E-cadherin over-expression in Raw 264.7 macrophage cell lines blunted the secretion of inflammatory mediators upon TLR engagement *in vitro*.

Doing so *in vivo*, macrophage E-cadherin could help to determine the outcome of typical Th2 cytokine-driven diseases like *Taenia crassiceps* helminth infection and allergic asthma, during which E-cadherin-expressing M_(IL-4/IL-13)_ macrophages are present. During the experimental mouse model of cysticercosis, infection with *Taenia crassiceps* evokes a Th1 response in the early phase of infection, which gradually switches to a Th2 response^35^,^36^. While Th2 responses are widely accepted to mediate protection against most helminths, a polarized STAT4-dependent, IL-12-mediated Th1 response and MIF-expressing inflammatory M1 macrophages are required to control *Taenia crassiceps* infections^37^,^38^,^39^. In accordance, STAT6-deficient mice, which lack AAMs and fail to induce Th2 responses, control *Taenia crassiceps* infections^22^,^40^. Based on the observations that E-cadherin has mainly inhibitory effects on pro-inflammatory signaling cascades in macrophages and other cell types, one would expect that Cdh1^Δ^ mice have more M1-polarized macrophages and thus clear *Taenia crassiceps* helminths more efficiently. However, the course of parasitemia as well as the leukocyte count and cellular composition was similar in the peritoneum of Cdh1^Δ^ and Cdh1^F/F^ infected mice, ruling out an important role of E-cadherin expression in macrophages during cysticercosis (Fig. 2). Additionally, the lack of macrophage E-cadherin expression during this strongly Th2 polarizing disease did not affect the basal macrophage activation state, nor the LPS(+IFNγ)-induced secretion of inflammatory factors (Fig. 3). In the latter case, an LPS+IFNγ-mediated downregulation of E-cadherin^6^ could explain why no differences were observed between *in vivo-*elicited Cdh1^Δ^ and Cdh1^F/F^ macrophages.

Allergic asthma is another prototypical Th2 cytokine-driven disease which strongly instructs E-cadherin expression in M_(IL-4/IL-13)_ macrophages. Yet, E-cadherin deletion in alveolar macrophages of Cdh1^Δ^ mice does not affect BAL cytokine levels and its cellular composition (Fig. 4). Based on the fact that E-cadherin is a cell adhesion molecule, one could hypothesize that E-cadherin^+^ alveolar macrophages could interact with E-cadherin^+^ epithelial cells in the lung, enabling their trapping and retention. However, since BAL macrophage counts were similar in Cdh1^Δ^ and Cdh1^F/F^ asthmatic mice, we have no evidence to support this hypothesis. Remarkably, B cell numbers were always lower in the BAL of ovalbumine-challenged Cdh1^Δ^ mice. While some B cell subsets are reported to express a nonclassical cadherin during B cell development^41^, we did not detect any E-cadherin-expressing CD11b-negative cells (including B cells) in the BAL of asthmatic mice (Fig. 2A). In addition, these B cells did not express the other E-cadherin ligands CD103 and KLRG1 (data not shown) and therefore it seems unlikely that the reduced B cell counts in the BAL of allergic Cdh1^Δ^ mice can be explained by a reduced trapping of those cells by E-cadherin-deficient alveolar macrophages. Hence, why B cell numbers are reduced during ovalbumin-induced allergic asthma in macrophage-specific E-cadherin-deficient mice remains unknown. Of note, while LysM-Cre x Cdh1^F/F^ mice are considered to be macrophage -specific E-cadherin-deficient mice, the DCs in the BAL of ovalbumine-challenged mice also displayed abrogated E-cadherin expression (Fig. 4A). Hence, alveolar DCs during allergic asthma display active lysozyme M promoters, which is in agreement with previous publications reporting lysozyme expression by some DC subsets^42^,^43^. In any case, deletion of E-cadherin in alveolar macrophages and DCs does not affect the degree of ovalbumin-induced experimental airway inflammation. Thus, while E-cadherin supresses inflammatory signaling in macrophages *in vitro*, these effects are clearly not strong enough to alter the overall macrophage activation status during polarized Th2 responses *in vivo*.

Overall, employing macrophage-specific E-cadherin-deficient mice, we demonstrate that E-cadherin in macrophages is largely unnecessary for *in vivo* granuloma formation, and for the regulation of Th2 responses. Irrespective of its *in vivo* redundancy, the E-cadherin/catenin complex offers a valuable tool to detect M_(IL-4/IL-13)_ macrophages *in vivo.*

## Methods

### Ethics Statement

The study was carried out in strict accordance with the recommendations in ‘Guidelines for the Use of Laboratory Animals in Research, Teaching and Testing’ of the International Council for Laboratory Animal Science. The permission of the local authorities has been given (accreditation N° LA1210220) and all animal work was approved by the appropriate committee (‘Ethische commisie voor dierproeven’) at the Vrije Universiteit Brussel (ethics committee protocol number 07-220-03).

### E-cadherin-overexpressing Raw264.7 cell lines

The generation of Raw264.7-E-cadherin transfectants were described previously^6^.

### Mice

To generate mice in which the E-cadherin gene was disrupted in macrophages, floxed Cdh1^F/F^ C57BL/6 mice (kind gift of Dr. J Jonkers, The Netherlands Cancer Institute, Amsterdam, The Netherlands^44^) were crossed with LysM-Cre C57BL/6 mice (Jackson Laboratory, Bar Harbour, Maine, USA^45^). Homozygous LysM-Cre^+/+^-Cdh1^F/F^ conditional KO (hereafter referred to as Cdh1^Δ^) mice were compared to LysM-Cre^−/−^-Cdh1^F/F^ littermate controls (hereafter referred to as Cdh1^F/F^). In Cdh1^Δ^ mice the IL-4-induced E-cadherin expression, either upon *in vitro* stimulation or during *in vivo* pathologies is ablated in >90% of the macrophages^6^.

### Disease models

To study cysticercosis, mice were inoculated intraperitoneally (ip) with 10 *Taenia crassiceps* cestodes, and peritoneal cells and helminths were collected at different time intervals post infection for further analysis^35^. To study the involvement of macrophage E-cadherin expression during granuloma formation in the lung, mice were injected intravenously (iv) with 5000 *Schistosoma mansoni* ova, were sacrificed 7 or 14 days later and the lungs were fixed and removed for further analysis (adapted from^24^).

To sensitize mice for allergic asthma, animals were injected ip at day 0 and 7 with 10 μg grade V chicken egg ovalbumin (OVA; Sigma) adsorbed on 1 mg Alum (Pierce, Rockford, IL) in PBS. Next, mice were challenged at day 14 and 15 (2× OVA, short term protocol) or at day 14, 15, 21 and 22 (4× OVA, long term protocol) for 30 min with aerosols, consisting of 1% grade III OVA in PBS^46^. 20 h after the last challenge, mice were sacrificed and broncho-alveolar lavage (BAL) was performed by PBS rinsing of the lungs. The BAL fluid (BALF) was centrifuged and supernatant and cell pellets were collected for further analysis.

### *In vitro* stimulation of macrophages

Bone marrow-derived macrophages (BMDM) were generated from naïve mice as detailed earlier^6^. Peritoneal macrophages from *Taenia crassiceps* infected mice were obtained by rinsing the peritoneum with PBS/10% sucrose. After 3 h culture, non-adherent cells were washed away and plastic-adherent macrophages were used for analysis. Macrophages were cultured for 24 h in RPMI1640 medium supplemented with 10% heat-inactivated FCS, 0.03% L-glutamine, 100 mg/mL streptomycin and 100 mg/mL penicillin, 1 mM nonessential amino acids, 1 mM sodium pyruvate (all from Invitrogen, Carlsbad, CA) and 0.02 mM 2-mercaptoethanol (Sigma-Aldrich, St. Louis, MO) in the presence of 0.1, 1 or 10 ng/ml *E. coli* LPS with or without 10 U/ml recombinant mouse IFN-γ or in the presence of 20 ng/ml recombinant mouse IL-4 (BD Bioscience).

### Cytokine, NO and arginase measurement

Cytokines in the macrophage culture supernatants were quantified with sandwich ELISAs for TNF (R&D Systems, Minneapolis, MN), IL-6, IL-12 and IL-10 (BD Pharmingen), in accordance to the suppliers’ protocols. Cytokine levels in BALF were measured by Bio-Plex (Bio-Rad, Hercules, CA). NO_2_^−^ in culture supernatants was quantified by a standard Griess reaction^47^. Arginase activity was measured as described earlier^48^.

### Flow cytometry and analysis of E-cadherin expression

Quantitative real-time PCR, Western blot and flow cytometry for E-cadherin mRNA and protein expression was performed as described earlier^6^. Gating on distinct immune cell types during the different disease models is shown in Figures S2 and S3. All antibodies are listed in Table 1. Data were acquired with a FACSCantoII (BD Biosciences) and analyzed using FlowJo (TreeStar, Ashland, OR).

### Schistosoma mansoni granuloma histopathology

For measurement of *Schistosoma mansoni* granulomas, lungs (5 mice per group) were inflated with a 1:1 PBS/Tissue-Tek OCT compound (Gentaur, Kampenhout, Belgium) mixture and stored at −20 °C. Next, 7 μm sections were prepared with a Leica CM1950 cryostat, stained with Hematoxilin-Eosin (HE) and acquired on Nikon Elipse 600 microscope using a Digital Sight DS-U2 and a 10×/0.25 (Ph1 DL WD 10.5), 20×/0.4 (Ph1 DL WD1.3) or a 40×/0.65 (Ph2 DL WD 0.57) objective lense (all from Nikon Instruments Inc., Lewisville, TX). At least 20 granulomas per mouse were analysed using ImageJ (National Institutes of Health). The volume of each granuloma was calculated as 4/3*Π*r^3^, the cross-sectional area was determined by ImageJ, the cell density was counted as the amount of cells per area of 10 μm^2^ and the percentage eosinophils in each cross-sectional area was evaluated manually.

### Statistics

Statistical significance was tested via the unpaired *t* test using GraphPad Prism 4 (GraphPad Software, San Diego, CA).

## Additional Information

**How to cite this article**: Van den Bossche, J. *et al.* E-cadherin expression in macrophages dampens their inflammatory responsiveness *in vitro*, but does not modulate M2-regulated pathologies *in vivo*. *Sci. Rep.* **5**, 12599; doi: 10.1038/srep12599 (2015).

## Supplementary Material

Supplementary Information

## Figures and Tables

**Table 1:** List of used antibodies.

**Marker/fluorophore**	**Clone**	**Isotype**	**Supplier**
rat IgG2a/pure isotype ctrl	NA/LE	rat IgG2a	BD Bioscience
E-cadherin/pure	ECCD2	rat IgG2a	Dr. M. Takeichi (University of Kyoto, Japan)
anti-rat Ig/PE/APC	polyclonal	goat Ig	BD Bioscience
CD16/CD32/pure Fc-Block	2.4G2	rat IgG2b	BD Bioscience
E-cadherin/pure (Western Blot)	36	mouse IgG2a	BD Bioscience
β-actin/pure (Western Blot)	AC-15	mouse IgG1	Abcam (Cambridge, UK)
CD11c/PerCp-Cy5.5	N418	hamster IgG	eBioscience (San Diego, CA)
Ly6c/AF647	ER-MP20	rat IgG2a	Serotec (Raleigh, NC)
F4/80/APC-Alexa Fluor 750	BM8	rat IgG2a	eBioscience
CD45.2/APC	104	mouse IgG2a	BD Bioscience
IA/IE/FITC	M5/114.15.2	rat IgG2b	eBioscience
Ly6G/FITC/PE	1A8	rat IgG2a	BD Bioscience
CCR3/FITC	83101	rat IgG2a	R&D Systems (San Jose, CA)
CD4/FITC	RM4-5	mouse IgG2a	eBioscience
CD8b/PE	Ly-3	rat IgG2b	BD Bioscience
CD19/PE	1D3	rat IgG2a	BD Bioscience
Siglec-F/PE	E50-2440	Rat IgG2a	BD Bioscience
CD11b-PE-Cy7	M1/70	rat IgG2b	BD Bioscience
CD3e/FITC	145-2C11	hamster IgG1	BD Bioscience
B220/PE	RA3-6B2	rat IgG2a	BD Bioscience
CD71/PE	C2	rat IgG1	BD Bioscience
CD273/PE	TY25	rat IgG2a	BBD Bioscience
CD206/APC	C068C2	rat IgG2a	Biolegend (San Diego, CA)
CD301/APC	ER-MP23	Rat IgG2a	Serotec
