# Supplementary material for: E-cadherin expression in macrophages dampens their inflammatory responsiveness in vitro, but does not modulate M2-regulated pathologies in vivo
Source: Sci Rep. 2015 Jul 31;5:12599. doi: 10.1038/srep12599 (PMC4521155; doi:10.1038/srep12599)
Supplement: Supplementary Information [file srep12599-s1.pdf]

SUPPLEMENTARY INFORMATION

E-cadherin expression in macrophages dampens their inflammatory responsiveness *in vitro*, but does not modulate M2-regulated pathologies *in vivo*

Jan Van den Bossche[1,2,*], Damya Laoui[1,2], Thomas Naessens[3], Hermelijn H. Smits[4], Cornelis H. Hokke[4], Benoît Stijlemans[1,2], Johan Grooten[3], Patrick De Baetselier[1,2], and Jo A. Van Ginderachter[1,2]

[1]Laboratory of Myeloid Cell Immunology, VIB, Brussels, Belgium

[2]Laboratory of Cellular and Molecular Immunology, Vrije Universiteit Brussel, Brussels, Belgium

* Current address: Department of Medical Biochemistry, Experimental Vascular Biology, Academic Medical Center, Amsterdam, The Netherlands

[3]Department of Biomedical Molecular Biology, Ghent University, Ghent, Belgium

[4]Department of Parasitology, Leiden University Medical Centre, Leiden, The Netherlands

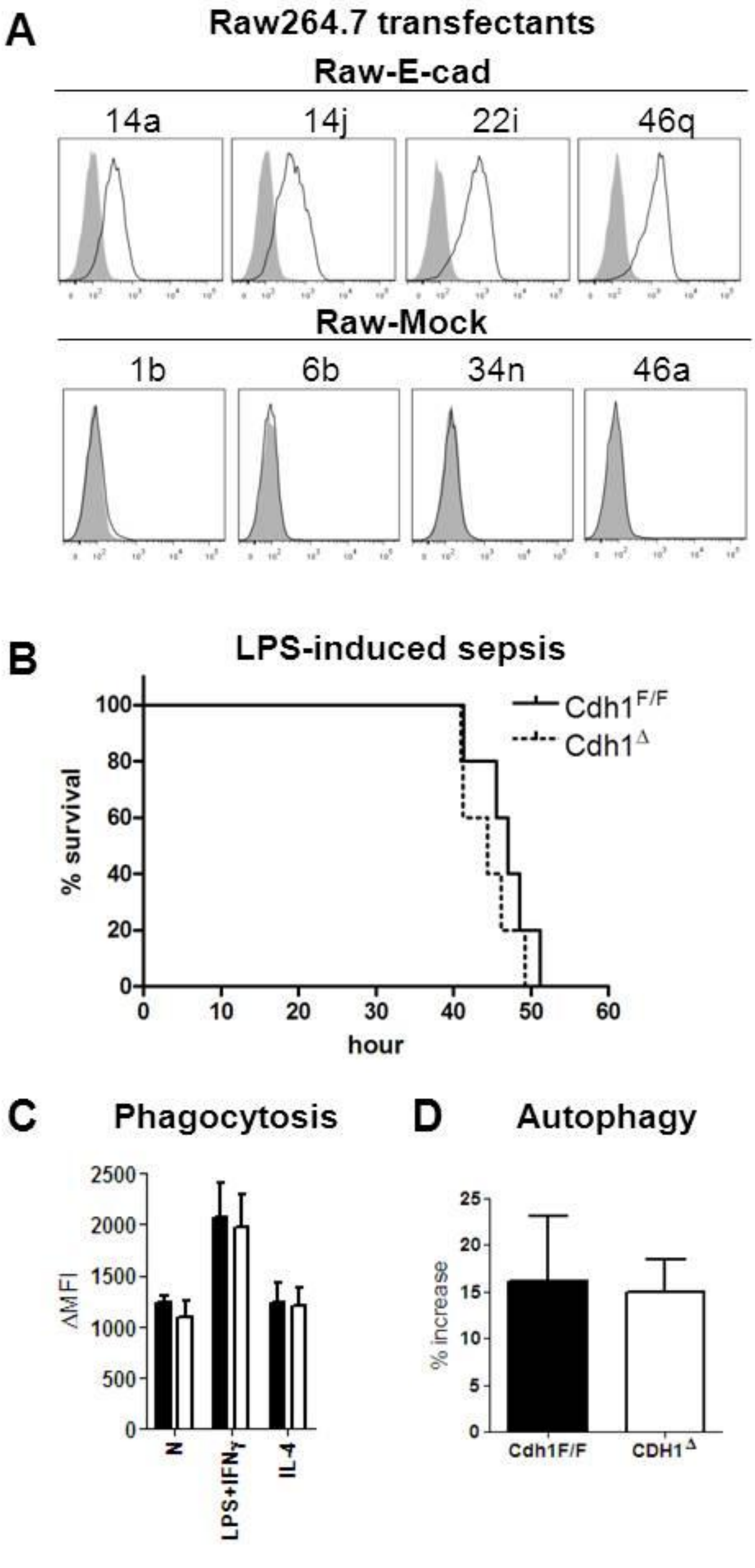
A
Raw264.7 transfectants
Raw-E-cad
14a
14j
22i
46q
Raw-Mock
1b
6b
34n
46a
B
LPS-induced sepsis
Cdh1F/F
Cdh1Δ
% survival
hour
C
Phagocytosis
ΔMFI
N
LPS+IFNγ
IL-4
D
Autophagy
% increase
Cdh1F/F
CDH1Δ

Figure S1

Figure S1. (A) Raw264.7 transfectants. Four independent E-cadherin over-expressing Raw264.7 transfectants and four E-cadherin-negative mock transfectants were generated as described earlier [6] and the E-cadherin surface expression of these clones was assessed by flow cytometry. **(B) LPS-induced sepsis.** An LPS-induced sepsis experiment was performed as typical M1 response *in vivo*. Hereto, 5 $Cdh1^{\Delta}$ and 5 $Cdh1^{F/F}$ mice (n=5) were injected with 12 mg/kg LPS (from *Escherichia coli* 0111:B4, Sigma) after which their survival was recorded. No differences were observed between the $Cdh1^{\Delta}$ mice and their WT ($Cdh1^{F/F}$) counterparts.**(C) Phagocytosis.** BMDM from naive $Cdh1^{\Delta}$ and $Cdh1^{F/F}$ mice (n=3) were treated with 10 ng/ml LPS + 10 U/ml IFNγ or with IL-4, or remained untreated for 24 h. Next, macrophages were cultured for 1 h at 4°C (control) or 37°C in the presence of green fluorescent latex beads (Polyscience, 1/5000 dilution). Latex bead uptake was assessed by flow cytometry and is shown as ΔMFI = [median fluorescence intensity] $_{37°C}$ - [median fluorescence intensity] $_{4°C}$. **(D) Autophagy.** $Cdh1^{\Delta}$ and $Cdh1^{F/F}$ BMDM were treated for 24 h with 500 nM rapamycin to induce autophagy or were left untreated. Next, a Cyto-ID Autophagy Detection Kit (Enzo Life Sciences) was used according to the manufacturer's protocol, followed by flow cytometry. Data show the rapamycin-induced increase in autophagy, compared to untreated macrophages.

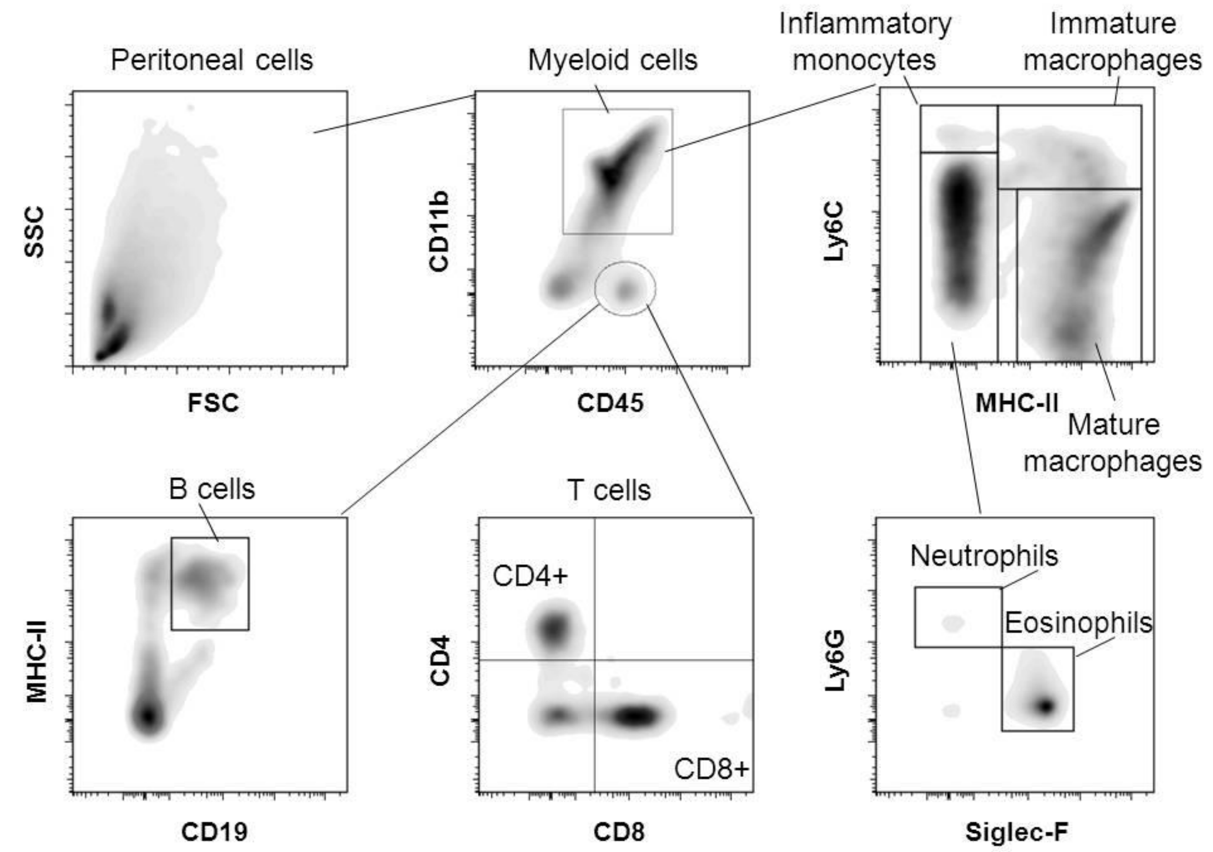

Figure S2. FACS gating strategy on freshly isolated peritoneal cells from *Taenia crassiceps* infected Cdh1$^{F/F}$ mice. Myeloid cells and other leukocytes were first separated based on CD11b/CD45 expression. Within the CD11b^{-}/CD45^{+} leukocyte gate, B cells were gated as MHC II^{high}/CD19^{+} and T cells were separated based on their differential CD4 and CD8 expression. Within the CD11b^{+} myeloid cell gate Ly6C/MHC II staining discriminates between Ly6C$^{high/}$MHC II^{-} inflammatory monocytes, Ly6C$^{high/}$MHC II^{+} immature macrophages and Ly6C$^{low\ to\ int/}$MHC II^{high} mature macrophages. Within the Ly6C$^{lo\ to\ int}$/MHC II^{-} gate, neutrophils were selected as Ly6G^{+} and eosinophils were gated as Siglec-F^{+}.

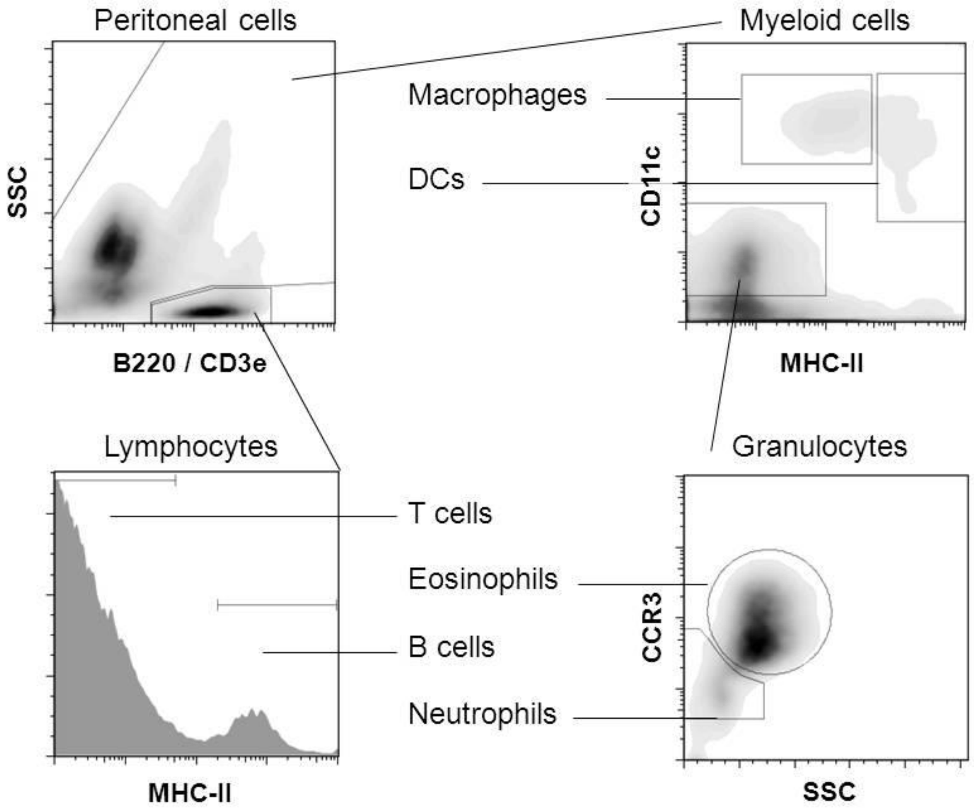

Figure S3. FACS gating strategy on cells of the bronchoalveolar lavage (BAL) of Cdh1$^{F/F}$ mice (4x OVA). First, lymphocytes were gated based on their B220/CD3 expression and low side scatter and further selected as B or T cells according to their high of low MHC II expression, respectively. Within the myeloid cell gate, macrophages were selected as CD11c^{hi}/MHC II^{int} and DCs as CD11c^{high}/MHC II^{high}. Within the CD11c^{-}/MHC II^{-} gate, eosinophils were gated as CCR3^{hi} and the remaining SSC^{int} population was marked as neutrophils.
